# Wastewater Minimization in Indirect Electrochemical Synthesis of Phenylacetaldehyde

**DOI:** 10.1100/tsw.2002.76

**Published:** 2002-01-05

**Authors:** Zhirong Sun, Xiang Hu, Ding Zhou

**Affiliations:** ^1^ School of Environmental and Energy Engineering, Beijing Polytechnic University, 100022, China; ^2^ School of Municipal and Environmental Engineering, Harbin Institute of Technology, 150006, China

**Keywords:** indirect electrochemical synthesis, phenylacetaldehyde, wastewater minimization

## Abstract

Wastewater minimization in phenylacetaldehyde production by using indirect electrochemical oxidation of phenylethane instead of the seriously polluting traditional chemical process is described in this paper. Results show that high current efficiency of Mn(III) and high yield of phenylacetaldehyde can be obtained at the same sulfuric acid concentration (60%). The electrolytic mediator can be recycled and there will be no waste discharged.

## INTRODUCTION

Phenylacetaldehyde is an intermediate widely used in the syntheses of fine chemicals such as phenylacetaldehyde dimethyl acetal. The traditional chemical process for its production has the disadvantage of being lengthy, low yield, and discharging large amounts of acid and basic effluents (see Fig. 1).

While the electrochemical method for its production may not only improve the yield of phenylacetaldehyde, it may also reduce the source of wastewater because of the possibility of the mediator recycling. The basic chemical equations are:

$$\mathrm{Mn(II)\text{-}e^- \xrightarrow{H_2SO_4} Mn(III)}$$

$$\mathrm{4Mn(III)+C_6H_5CH_2CH_3+H_2O \xrightarrow{H_2SO_4} 4Mn(II)+C_6H_5CH_2CHO+4H^+}$$

In recent years much work has been done to reduce the discharge of wastewater and improve the yield of benzaldehyde in electrochemical production processes[1,2,3,4,5]. Few reports have been shown about the production of phenylacetaldehyde by electrochemical synthesis with less pollution. In order to realize the mediator Mn(III)/Mn(II) used circularly without discharge in the

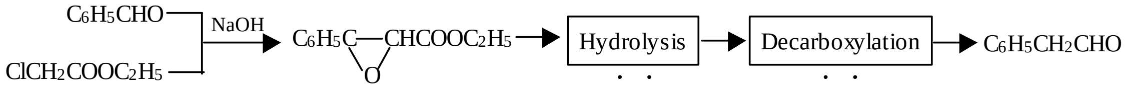

**FIGURE 1**. A schematic drawing of the traditional chemical production process for phenylacetaldehyde; "··"marks the points of waste discharge.

electrosynthesis of phenylacetaldehyde, electrolysis of Mn(II) to Mn(III), an oxidation of phenylethane to phenylacetaldehyde should be carried out in the same $H_2SO_4$ concentration. In this paper, the optimum condition of Mn(III) oxidizing phenylethane has been studied and the improvement of the current efficiency of electrolytic production of Mn(III) in the same $H_2SO_4$ concentration has also been discussed.

## EXPERIMENT

### Materials and Instruments

- $MnSO_4{\cdot}H_2O$ AR, concentrated $H_2SO_4$ (92%) AR, 0.2 mol/l standard ferrous ammonium sulfate solution, phenylethane AR, $Na_2CO_3$ (10%), NaOH AR, CTAB AR, self-made Pb-Sb-As electrode.
- Electric stirrer (D25-2F), voltage stabilizer (WYJ-50V, 3A), automatic electronic balance (Japan), Tachometer (Switzerland).

### Experimental Procedures

100 ml 60% $H_2SO_4$ solution and 1 mol/l $MnSO_4$ are added into the electrolytic cell. Self-made Pb-Sb-As alloy is used as anode and cathode; the electrolysis is carried on under stirring. After electrolysis, the electrolyte is moved to a three-necked flask and oxidizes phenylethane to phenylacetaldehyde under strong stirring.

### Analyses of Mn(III) and Phenylacetaldehyde

The concentration of Mn(III) is determined by titration with standard ferrous ammonium sulfate solution. The current efficiency η is:

$$\eta\% = \frac{[Mn(III)] \bullet V \bullet F}{I \bullet t} \times 100\%$$

where η: current efficiency; [Mn(III)]: concentration of Mn(III), mol/l; V: volume of electrolyte, l; F: Faraday constant; I: current strength, A; t: time of electrolysis, h.

Phenylacetaldehyde is analyzed by the method of $NaHSO_3$ addition.

## RESULTS AND DISCUSSION

### Optimization of the Conditions of Mn(III) Oxidizing Phenylethane to Phenylacetaldehyde in 60% $H_2SO_4$

In order to obtain higher yield of phenylacetaldehyde, orthogonal tests of Mn(III) oxidizing phenylethane are carried out (see Table 1).

**TABLE 1**
**Orthogonal Tests ($L_{16}[4^5]$) of Mn(III) Oxidizing Phenylethane**

| Number | $H_2SO_4$ Concentration (%) | Temperature (°C) | CTAB | Mn(III): Phenylethane (mole ratio) | Yield of Phenylacet-aldehyde (%) |
|---|---|---|---|---|---|
| 1 | (1) 40 | (1) 40 | (1) 0.1 | (1) 1:2 | 0 (15 h) |
| 2 | (1) 40 | (2) 50 | (2) 0.0 | (2) 1:3 | 27.8 |
| 3 | (1) 40 | (3) 60 | (3) 0.2 | (3) 1:4 | 33.8 |
| 4 | (1) 40 | (4) 70 | (4) 0.3 | (4) 1:5 | 10.0 |
| 5 | (2) 50 | (1) 40 | (2) 0.0 | (3) 1:4 | 43.4 |
| 6 | (2) 50 | (2) 50 | (1) 0.1 | (4) 1:5 | 54.1 |
| 7 | (2) 50 | (3) 60 | (4) 0.3 | (1) 1:2 | 31.2 |
| 8 | (2) 50 | (4) 70 | (3) 0.2 | (2) 1:3 | 32.0 |
| 9 | (3) 60 | (1) 40 | (3) 0.2 | (4) 1:5 | 38.2 |
| 10 | (3) 60 | (2) 50 | (4) 0.3 | (3) 1:4 | 33.0 |
| 11 | (3) 60 | (3) 60 | (1) 0.1 | (2) 1:3 | 55.2 |
| 12 | (3) 60 | (4) 70 | (2) 0.0 | (1) 1:2 | 52.0 |
| 13 | (4) 70 | (1) 40 | (4) 0.3 | (2) 1:3 | 22.0 |
| 14 | (4) 70 | (2) 50 | (3) 0.2 | (1) 1:2 | 21.4 |
| 15 | (4) 70 | (3) 60 | (2) 0.0 | (4) 1:5 | 45.8 |
| 16 | (4) 70 | (4) 70 | (1) 0.1 | (3) 1:4 | 32.5 |
| $r_{1j}$ | 17.9 | 25.9 | 35.5 | 26.2 | |
| $r_{2j}$ | 40.2 | 34.1 | 42.3 | 34.3 | |
| $r_{3j}$ | 44.6 | 41.5 | 31.4 | 35.7 | |
| $r_{4j}$ | 30.4 | 32.6 | 24.1 | 37.0 | |
| $R_j$ | 26.7 | 15.6 | 18.2 | 10.8 | |

CTAB: a kind of phase transfer catalyst.

Table 1 shows that $H_2SO_4$ concentration and CTAB have great effects on yield of phenylacetaldehyde. The optimum condition of Mn(III) oxidizing phenylethane to phenylacetaldehyde is: $H_2SO_4$ concentration, 60%; temperature, 60ºC; mole ratio of Mn(III) to phenylethane, 1:3–1:5; strong stirring; and the yield of phenylacetaldehyde will be 58% under this condition.

The optimum $H_2SO_4$ concentration for Mn(III) oxidizing phenylethane to phenylacetaldehyde has been obtained as 60%, so the optimum electrolytic condition is studied in the same $H_2SO_4$ concentration.

## Optimization of the Conditions of Electrolyzing $MnSO_4$ to Mn(III)

Current efficiency of heterogeneous electrochemical oxidation of Mn(II) to Mn(III) is closely related to the electrolytic conditions. The effects of $MnSO_4$ concentration, electrolytic temperature, current density, electrolytic time, and stirring speed on the current efficiency are discussed in this paper. Experimental results show that the optimum parameters for heterogeneous electrolysis of $MnSO_4$ in 60% $H_2SO_4$ using both Pb-Sb-As alloy as anode and cathode are: $MnSO_4$ concentration, 1.0 mol/l; current density, 60 mA/cm$^2$; temperature, 60ºC; electrolytic time, 2 h; stirring speed, 1000 r/min. Under the above conditions, a current efficiency up to 76% can be obtained, which is a little less than that of 80% obtained in the 40% $H_2SO_4$ electrolytic solution.

## Recycling of the Mediator and Sulfuric Acid Solution

In order to exert the characteristics of the technology of phenylethane to phenylacetaldehyde without waste, $MnSO_4$ and sulfuric acid solution should be used circularly. But experimental

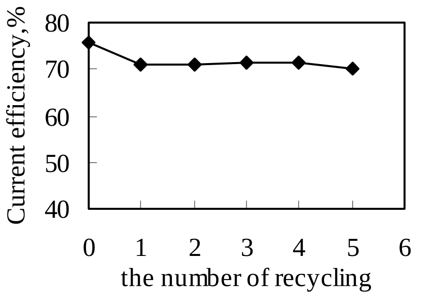

**FIGURE 2**. The effect of the recycling number on current efficiency.

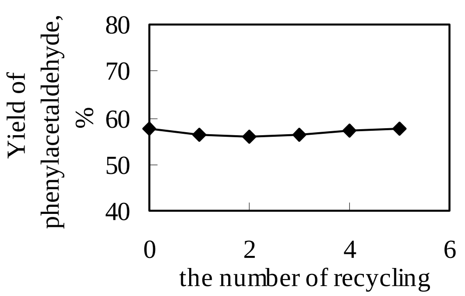

**FIGURE 3**. The effect of the recycling number on yield of phenylacetaldehyde.

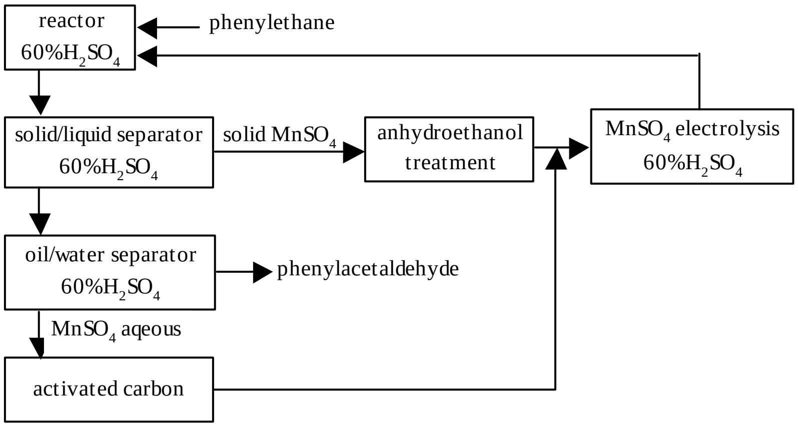

**FIGURE 4**. Flow diagram of the process of heterogeneous indirect electroproduction of phenylacetaldehyde in 60% $H_2SO_4$ with recycling of the mediator.

results have shown that if the sulfuric acid solution and solid $MnSO_4$ are recycled, and used as they are obtained from oil/water separation without any purification, current efficiency will fall greatly. This is probably due to the small amounts of organic impurities produced in the oxidation of phenylethane by Mn(III) and remaining in the sulfuric acid solution and solid $MnSO_4$. These impurities either will react on the anode or cathode or will further react with Mn(III) causing the fall in current efficiency.

Experimental results show that after the sulfuric acid solution is treated with activated carbon A, solid $MnSO_4$ is washed with anhydroethanol; the current efficiency during recycling processes can remain in a constant level, approximately above 70% (see Fig. 2). The yield of phenylacetaldehyde can remain about 57% (see Fig. 3). So the recycling of mediator in indirect electrosynthesis of phenylacetaldehyde is realized and there is no waste to be discharged (see Fig. 4).

## CONCLUSION

Electrosynthesis of phenylacetaldehyde with less pollution is studied in this paper. Both processes of heterogeneous electrolysis of $MnSO_4$ and synthesis of phenylacetaldehyde by oxidation of Mn(III) can be carried out separately in the same concentration of sulfuric acid (60% $H_2SO_4$) with high current efficiency and high yield of phenylacetaldehyde. The organic impurities in sulfuric acid solution and solid $MnSO_4$ causing the fall in current efficiency are removed effectively by activated carbon A and anhydroethanol, respectively. So the cleaner technology of the mediator recycling of electrosynthesis of phenylacetaldehyde can be realized and it may be used in production in the future.
